# A Case Series of Steroid‐Responsive Encephalopathy Associated With Autoimmune Thyroiditis (SREAT) With Atypical Thyroid Lab and Imaging Findings

**DOI:** 10.1155/crnm/8314930

**Published:** 2026-05-12

**Authors:** Mariah S. Johnson, Maxim Petrovsky, Rachel Rinehart Mimbella, Adnan Ecizi, Madelynn Paul, Anna Hohler

**Affiliations:** ^1^ Tufts University School of Medicine, Boston, Massachusetts, USA, tufts.edu; ^2^ Boston University Chobanian & Avedisian School of Medicine, Boston, Massachusetts, USA; ^3^ Department of Psychiatry, Boston Medical Center, Brighton, Massachusetts, USA, bu.edu; ^4^ Department of Internal Medicine, Boston Medical Center, Brighton, Massachusetts, USA, bu.edu; ^5^ Department of Neurology, Boston Medical Center, Brighton, Massachusetts, USA, bu.edu

**Keywords:** autoimmune encephalopathy, case series, corticosteroids, steroid-responsive encephalopathy associated with autoimmune thyroiditis, thyroid antibodies

## Abstract

Steroid‐responsive encephalopathy associated with autoimmune thyroiditis (SREAT) is a rare, potentially reversible neuropsychiatric syndrome linked to autoimmune thyroid disease. However, its clinical heterogeneity may stray from the most accepted diagnostic criteria often leading to a delay in its recognition. In this paper, we describe two patients who presented with subacute encephalopathy for which the diagnosis of SREAT was established after the detection of elevated antithyroid antibody titers and the exclusion of alternative etiologies. Notably, both cases exhibited atypical features, namely CSF lymphocytic pleocytosis and leptomeningeal enhancement in Case 1 and a complex differential confounded by multiple comorbidities in Case 2. Treatment strategies included corticosteroid‐based immunosuppression, with one patient also receiving intravenous immunoglobulin and thyroid hormone replacement. Both patients demonstrated substantial neurological improvement following therapy, underscoring the reversibility of this condition when appropriately treated. Together, these cases highlight the importance of maintaining diagnostic suspicion for SREAT in patients with unexplained encephalopathy, even in the absence of overt thyroid dysfunction, atypical lab and imaging findings, and a complex past medical history. Early recognition and initiation of immunotherapy can significantly alter outcomes and prevent prolonged morbidity. By presenting these cases, we aim to add to the limited body of literature on SREAT and emphasize the critical role of maintaining suspicion for SREAT in atypical cases of probable autoimmune encephalitis to facilitate timely diagnosis and intervention in optimizing patient recovery.

## 1. Introduction

Steroid‐responsive encephalopathy associated with autoimmune thyroiditis (SREAT), previously known as Hashimoto’s encephalopathy, is a rare and elusive neurological disorder first described in 1966 [1, 2]. The estimated prevalence of SREAT is approximately 2.1 per 100,000, which is likely underreported and shows a striking female predominance (female‐to‐male ratio of about 4:1) [3–5]. Despite ongoing research, SREAT remains poorly understood. SREAT is characterized by the presence of elevated antithyroid antibodies and can occur in any thyroid state; however, most patients are euthyroid at diagnosis [2, 4, 6–8]. While elevated antithyroid antibodies (especially anti‐TPO and anti‐TG) are almost universally present, this lab finding lacks disease specificity. Further, there is no evident correlation between the clinical manifestation of SREAT and the elevation of antithyroid antibodies [7–9]. Graus et al. proposed diagnostic criteria for autoantibody‐negative but probable autoimmune encephalitis, which includes SREAT, in order to facilitate timely appropriate treatment without the need to wait for antibody test results, yet this condition continues to be frequently misdiagnosed and mistaken for other conditions including viral encephalitis, Creutzfeldt–Jakob disease, or neurodegenerative dementia [10]. This is in part due to its clinical presentation being highly variable and mimicking a range of other conditions such as stroke, dementia, encephalitis, epilepsy, or primary psychiatric disorders [1, 4, 6, 8–11]. However, misdiagnosis may also be in the context of inappropriate use of diagnostic criteria for antibody‐negative disease. To address this issue, Graus and Dalmau performed an analysis of the most common causes of SREAT misdiagnosis, proposing a solution reliant on careful application of criteria and appropriate antibody testing [12].

There are two broadly accepted clinical phenotypes. One is a vasculitic or “stroke‐like type,” which is distinct from a true vasculitis but shares some phenotypic features such as focal neurological deficits. The other, and more common, phenotype is a diffuse, progressive type characterized by acute or subacute altered mental status, seizures, myoclonus, tremor, aphasia, and gait ataxia [2, 5, 7–9]. The underlying pathogenesis remains unclear, with proposed mechanisms including autoimmune reactions against shared CNS–thyroid antigens, autoimmune vasculitis, and toxic effects of thyrotropin‐releasing hormone (TRH). Disease activity appears to be immune‐mediated rather than directly related to thyroid hormone dysfunction, as antibody titers and thyroid hormone levels do not consistently correlate with disease severity. Additionally, most patients respond well to immunosuppressive therapy, further suggesting an immune‐mediated process [2, 4–6, 8, 9].

Diagnostic findings in SREAT are heterogeneous, reinforcing its classification as a diagnosis of exclusion. Cerebrospinal fluid (CSF) analysis frequently shows elevated protein without pleocytosis, while neuroimaging is often normal or shows nonspecific changes. Intrathecal synthesis of antithyroid antibodies may be detected, although its clinical significance is uncertain [2, 4, 5, 8–11]. Additionally, a case series and literature review performed by Chaudhuri et al. found EEG abnormalities observed in over 90% of reported cases of SREAT. In addition to diagnostic complexity, this condition also lacks a formal approach to treatment that needs to be defined.

A hallmark feature aiding diagnosis is a rapid and often dramatic clinical improvement with corticosteroid treatment [13]. Notably, Chong et al. performed a literature review which found improvement in 96% of cases treated with glucocorticoids [6]. Additional immunosuppressive therapies, including intravenous immunoglobulin (IVIG), plasmapheresis, rituximab, methotrexate, and mycophenolate, have also shown benefit. One report suggests that antibody titers may decrease in parallel with clinical improvement, raising the possibility of using them as markers of disease activity; however, this has not been proven in further studies [9].

Given the heterogeneity of possible SREAT presentations, criteria for probable antibody‐negative autoimmune encephalitis remain broad: (1) rapid progression (< 3 months) of working memory deficits, altered mental status, or psychiatric symptoms; (2) exclusion of well‐defined syndromes of autoimmune encephalitis; (3) absence of well‐characterized autoantibodies in serum and CSF, and at least two of the following: MRI abnormalities suggesting autoimmune encephalitis, CSF pleocytosis, CSF‐specific oligoclonal bands, or elevated CSF IgG index, brain biopsy showing inflammatory infiltrates, and excluding other disorders (e.g., vasculitis or tumor); and (4) reasonable exclusion of alternative causes. However, it is crucial to note that these are not all‐inclusive, standalone criteria. Instead, their fulfillment only suggests antibody‐negative autoimmune encephalitis be considered as a diagnosis [12].

Overall, SREAT remains a rare and heterogeneous condition, posing significant diagnostic challenges that can delay timely intervention. In this study, we retrospectively review the clinical features, diagnostic workup, treatment response, and outcomes of two patients diagnosed with SREAT while hospitalized. By systematically characterizing this small cohort, we aim to contribute to a clearer understanding of SREAT and emphasize that early recognition and prompt immunosuppressive therapy can lead to favorable clinical outcomes.

## 2. Case 1

A 58‐year‐old female with a past medical history of juvenile idiopathic arthritis, celiac disease, and hypothyroidism on levothyroxine presented to the emergency department from an outside hospital with one month of progressively worsening neurological symptoms, including dizziness, imbalance, left facial droop, word‐finding difficulties, and right‐sided headache. Two days prior to admission, her condition rapidly deteriorated, resulting in severe somnolence and mutism. The initial presentation was most concerning for encephalitis, metabolic encephalopathy, or stroke. Stroke was initially included in the differential as a cannot‐miss diagnosis given the acute onset of focal deficits. However, the distribution of symptoms spanning posterior and bilateral anterior territories made a single vascular event less likely and favored a diffuse, multifocal process.

**FIGURE 1:**
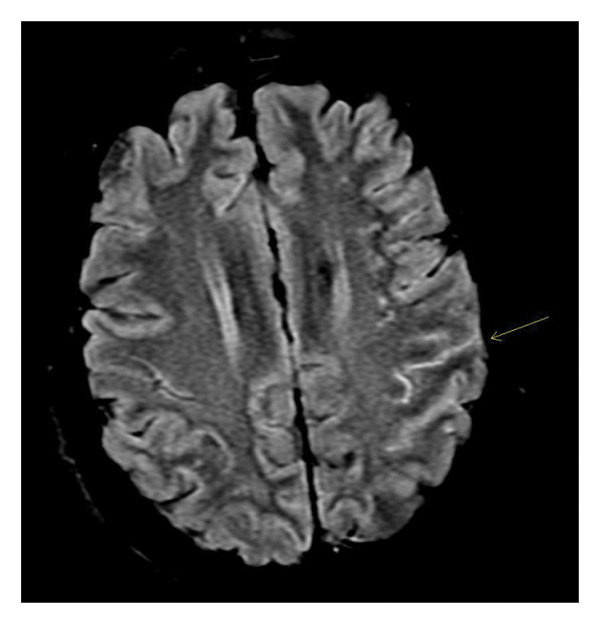
MRI brain, axial T2 FLAIR sequence (calvaria not visible as image brightness was adjusted to optimize visualization of leptomeningeal signal).

Initial laboratory investigations revealed leukocytosis (17.5 × 10^3^/μL), elevated inflammatory markers (ESR 94 mm/hr; CRP 4.54 mg/dL), mildly elevated TSH (6.40 µIU/mL), and normal free T4 (1.64 ng/dL). Noncontrast CT head and CTA of the head and neck were negative for acute pathology, and urine toxicology was unremarkable. Brain MRI with and without contrast demonstrated mild, diffuse leptomeningeal enhancement, raising concern for meningitis and prompting lumbar puncture for CSF analysis (Figure 1). Specifically, the axial FLAIR images revealed increased signal within parietal sulci, which may be seen in autoimmune meningoencephalitis. CSF studies showed lymphocytic pleocytosis (WBC 414 cells/μL; 74% lymphocytes), elevated protein (162 mg/dL), normal glucose (50 mg/dL), and negative Gram stain, fungal culture, and CSF culture. These results were most concerning for viral encephalitis, particularly HSV encephalitis. However, per infectious diseases consultants, HSV encephalitis would have caused a more rapid deterioration without treatment and, while variable, most typically demonstrates a T2‐FLAIR hyperintensity in the mesial temporal lobes on MRI rather than the leptomeningeal enhancement pattern seen here. Nonetheless, they suggested starting empiric acyclovir on hospital day 2 until HSV PCR from CSF returned. IVIG was started at the same time for a 5‐day course, given concern for autoimmune encephalopathy. Ceftriaxone 1 g was also initiated for a GBS‐positive urine result from an outside hospital and was increased to 2 g to empirically cover Lyme disease.

**FIGURE 2:**
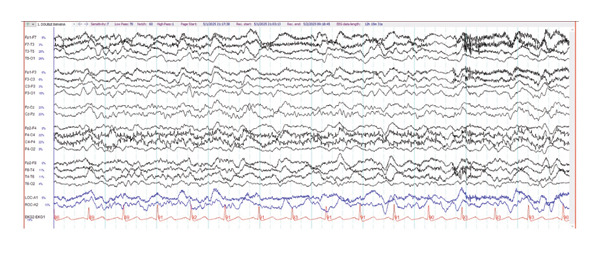
Continuous EEG sample from case 1.

On hospital day 3, IVIG was discontinued after two doses in favor of starting methylprednisolone 1 g daily, given increased suspicion of SREAT due to continuous EEG remarkable for slow, disorganized background rhythms (Figure 2), positive thyroglobulin and thyroid peroxidase antibodies (57.4 and 84 IU/mL, respectively), and a clinical presentation that continued to be atypical for viral meningitis. Levetiracetam was also initiated given abnormal cEEG findings. By hospital day 5 (Day 3 of the methylprednisolone course), the patient had already exhibited significant improvements in sociocognitive ability, including increased interactiveness and the ability to follow simple commands. However, she remained disoriented to time and place with mild anisocoria. The following day, HSV PCR from CSF returned negative and, after discussion with ID, acyclovir was discontinued given a low pretest probability of HSV encephalitis and remarkable improvement on steroids. On hospital day 7, the patient was noted to be more oriented and was able to recall memories prior to hospitalization. However, she continued to demonstrate some word‐finding difficulties and a mild simple kinetic tremor, and IVIG was restarted. The patient was discharged on hospital day 9 after completing a 5‐day course of IVIG, a 5‐day course of high‐dose IV methylprednisolone, and a transition to prednisone 60 mg PO to be tapered by an outpatient neurologist. By discharge, she had exhibited a rapid and near‐complete return to her neurological baseline.

Extensive workup for alternative etiologies was performed during her hospital course and was largely unremarkable. Workup from the outside hospital from which she was transferred included CSF Lyme, CSF meningoencephalitis panel, CSF VDRL, CSF AFB smear culture, MS1 panel, CSF oligoclonal bands, serum HSV1/2, serum WNV, serum VZV DNA QL PCR, Lyme IgM/IgG, HIV 1/2 Ab screen, syphilis screen, and HCV RNA Qn w/R. All tests were negative. At this hospital, further testing was performed including CSF LP ACE protein, paraneoplastic panel, ENC2 panel, PAC1, myelin basic protein, serum EEE serology (sent to Mayo), serum Powassan panel (sent to Mayo), serum Lyme total antibody, serum WNV IgM, serum WNV IgG, serum 14‐3‐3, serum RPR, serum Treponema antibody, ANA, SS‐A antibody, SS‐B antibody, MAG antibody, islet cell Ag 512 antibody, anti‐GAD 65 antibody, and insulin antibody. Of these, only serum WNV IgG and anti‐GAD 65 antibodies (135 U/mL) were positive. Paraneoplastic encephalitis workup via CT scans of the chest, abdomen, and pelvis was also negative.

## 3. Case 2

A 64‐year‐old female with a past medical history significant for alcohol use disorder, depression, migraines, multiple falls with intracranial bleeding, gait instability, and Hashimoto’s thyroiditis presented by ambulance after she was found down at home and was admitted for acute‐on‐chronic subdural hematoma and traumatic subarachnoid hemorrhage. On initial examination, she was disoriented, confused, and unable to provide any meaningful history of present illness.

**FIGURE 3:**
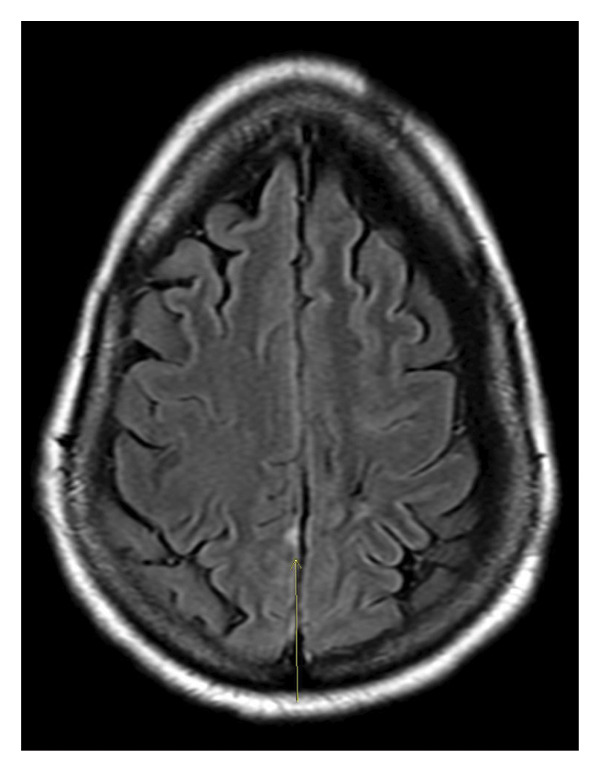
MRI brain, axial T2 FLAIR sequence (image brightness adjusted to optimize visualization).

**FIGURE 4:**
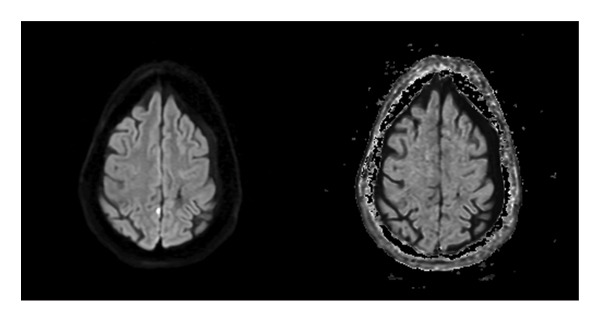
MRI brain, axial diffusion‐weighted (b1000) with corresponding ADC map.

**FIGURE 5:**
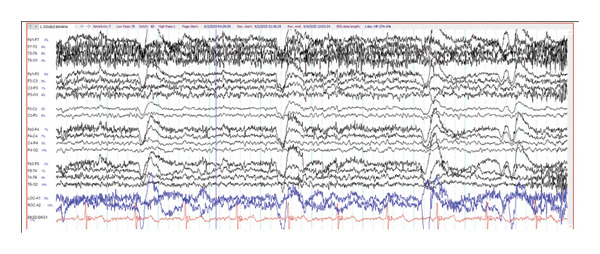
Continuous EEG sample from case 2.

Initial noncontrast CT scan showed a right frontoparietal acute‐on‐chronic subdural hematoma measuring up to 5.6 mm, a left frontoparietal chronic subdural hematoma measuring up to 4.6 mm, traumatic subarachnoid hemorrhage, and minimal intraventricular hemorrhage of the bilateral lateral ventricles without midline shift. Due to the small size of the acute SDH, she did not require surgical intervention. She was also found to have a chronic lacunar infarct in the left corona radiata and periventricular white matter hypodensities compatible with chronic small vessel ischemia. Brain MRI with and without contrast showed small foci of diffusion restriction of the medial right parietal lobe and the lateral right temporal lobe, consistent with small acute infarcts (Figures 3 and 4). No acute intraparenchymal hemorrhage was found. After discussion at a radiology conference, it was believed that the diffusion restriction was more likely due to acute traumatic injury rather than an acute ischemic or embolic stroke. Hyperreflexia and clonus were present on physical examination, prompting an MRI of the cervical spine, which showed multilevel cervical spondylosis with mild‐to‐moderate canal stenosis and severe foraminal stenosis without a visualized cord signal abnormality. These findings raised the possibility of compressive cervical myelopathy as a contributor, though the absence of T2 signal change in the medulla precluded a definitive imaging‐based diagnosis. TTE incidentally identified an atrial wall shunt suspicious for a patent foramen ovale. Forty‐eight‐h continuous EEG demonstrated an unevenly modulated and poorly organized pattern suggestive of mild encephalopathy (Figure 5). Initial laboratory results were generally unrevealing, with a WBC of 6.5 × 10^3^/μL, BUN/creatinine ratio of 18.3, calcium of 10.1 mg/dL, AST 97 H U/L, ALT 47 U/L, and total creatine kinase 2368 U/L.

Given her history of alcohol use disorder, the leading differential diagnosis was toxic metabolic encephalopathy superimposed on a subdural hematoma and subarachnoid hemorrhage secondary to trauma rather than emboli through the PFO. Under this assumption, she was managed with intravenous thiamine 500 mg three times daily for 4 days followed by 500 mg IV once daily for 6 days, and then 100 mg PO daily. She was also prescribed levetiracetam for seizure prophylaxis and supportive care. However, she continued to be disoriented, and neurological deficits seen on physical examinations persisted despite metabolic correction. Subsequent encephalopathy workup was significant for a TSH (73.3 µIU/mL), T4 (0.39 ng/dL), positive serum thyroglobulin (7.5 IU/mL), and TPO antibodies (185 IU/mL), raising suspicion for SREAT. Treatment included pulse doses of corticosteroids beginning with a 40 mg dose of prednisone for 5 days starting on hospital day 10, with nearly immediate cognitive improvement. Prednisone 40 mg daily was continued for one week followed by a slow taper decreasing by 10 mg prednisone (30 mg daily in the first week, 20 mg in the second week, and then 10 mg in the third week). On this regimen, the patient demonstrated continued significant improvement in functional and cognitive capacity. Her hypothyroidism was also managed with daily levothyroxine 112 μg. She was discharged on hospital day 17, 7 days after the initiation of steroids. Notably, the patient was medically ready to leave for several days prior to discharge but remained admitted due to issues with discharge and follow‐up disposition.

## 4. Discussion

SREAT remains a diagnostically challenging and clinically heterogeneous disorder that can present with a wide range of neurological and psychiatric symptoms, often mimicking more common conditions such as stroke, autoimmune encephalitis, or toxic‐metabolic encephalopathy [2, 4, 5, 8–10]. Here, we present two cases that illustrate unusual clinical manifestations, the importance of maintaining diagnostic suspicion in complex scenarios, and the critical role of systematic evaluation and timely immunosuppressive therapy in achieving favorable outcomes. Both demonstrate that SREAT has a highly variable clinical presentation that occurs across thyroid states, even among patients with the more common diffuse progressive phenotype. Importantly, although both cases have atypical features, they each satisfy the three prerequisites proposed by Graus and Dalmau to appropriately admit the diagnosis of SREAT onto the differential [12].

### 4.1. Review of Cases

In the first case, the patient presented in a subclinical hypothyroid state with an insidious cognitive decline that rapidly progressed to severe somnolence and mutism, leading up to her hospital presentation. This pattern is most consistent with the diffuse progressive phenotype of SREAT and fulfills the first two diagnostic criteria per Graus et al. Although initial neuroimaging raised concern for meningitis due to mild diffuse leptomeningeal enhancement and lymphocytic pleocytosis with elevated protein on CSF analysis, an extensive infectious and autoimmune workup ultimately proved negative. While this patient had positive serum anti–GAD‐65 antibody, GAD‐65–related disease could be reasonably excluded given negative CSF GAD‐65 antibody and the absence of clinical features (i.e., Stiff–Person Syndrome, limbic encephalitis, and rhomboencephalitis) [14]. Additionally, diagnosis of these conditions requires high anti–GAD‐65 antibody titers, typically > 2000 U/mL, while this patient’s titers were only elevated to 135 U/mL [15]. The subsequent discovery of elevated CSF antithyroglobulin and thyroid peroxidase antibodies, combined with the exclusion of alternative diagnoses, led to suspicion of SREAT and prompted the administration of IVIG and corticosteroids five and six days after presentation, respectively. There was rapid clinical improvement to near‐baseline following corticosteroid therapy, confirming the diagnosis of SREAT. This case highlights a classic feature described in the literature: SREAT remains a diagnosis of exclusion, often confirmed retrospectively through a dramatic therapeutic response. However, two findings in this case stray from the typical workup results associated with the diagnosis of SREAT and even contradict two diagnostic criteria defined by Graus et al.: leptomeningeal enhancement on MRI and lymphocytic pleocytosis in CSF analysis. These two features are discussed in further detail in the following section.

The second case demonstrates significant diagnostic complexity, as the patient had multiple comorbidities including alcohol use disorder, chronic subdural hematomas, prior traumatic brain injury, and severe cervical myelopathy. The clinical picture included mild encephalopathy after being found down in her home following a fall associated with clonus and hyperreflexia on examination, consistent with the first diagnostic criteria per Gaus et al. Initially, this presentation was attributed to toxic‐metabolic encephalopathy superimposed on traumatic brain injuries. Brain MRI and CT scan findings were most likely secondary to shear trauma from multiple falls rather than small vessel disease. In light of this, further workup for vasculitis was not pursued. However, the persistence of disorientation and focal neurological deficits despite metabolic correction prompted further evaluation. Profound hypothyroidism alongside positive antithyroid antibodies shifted clinical suspicion toward SREAT. Treatment with high‐dose intravenous corticosteroids and thyroid hormone replacement led to noticeable yet incomplete improvement, lending further support to the diagnosis of SREAT.

Both cases demonstrated marked clinical improvement following immunosuppressive therapy, reinforcing the view of SREAT as primarily immune‐mediated rather than directly driven by thyroid dysfunction. Of note, neither patient returned to baseline during their hospital stay, and they were discharged with a tapering course of steroids to continue at home. Both patients had returned to their baseline functioning levels at the time of follow‐up 2 weeks after discharge.

### 4.2. What Makes These Cases Atypical

Together, these two cases demonstrate several themes consistent with prior research while also containing atypical elements that may broaden possible presentations of SREAT. In the first case, the patient demonstrated leptomeningeal enhancement on neuroimaging, which led to an initial primary differential of HSV2 meningitis. This is a feature that is rarely seen in SREAT, which is known to typically demonstrate normal or nonspecific brain MRI findings. Though rare, there have been case reports of SREAT that presented with parenchymal and meningeal abnormalities on MRI that may even persist for several years following treatment [10]. Mechanistically, leptomeningeal enhancement may be due to the more persistent, diffuse perivascular lymphocyte infiltration seen in SREAT, leading to inflammation beyond the parenchyma [16].

The first case also demonstrated CSF pleocytosis, an atypical feature in SREAT. This likely reflects an immune‐mediated disruption of the blood–brain barrier, consistent with the theorized autoimmune pathogenesis of SREAT coupled to the coexisting autoimmune thyroiditis [17]. This is corroborated by several case studies and pathological reports that demonstrate a lymphocyte‐driven inflammatory process with corresponding CSF pleocytosis, potentially driven by an autoimmune reaction to antigens shared by the thyroid gland or to non–thyroid‐related antigens [16, 18, 19]. Pfeuffer et al. identified lymphocytic pleocytosis in almost all 22 patients included in their study. Their analysis demonstrated the continued presence of activated CD4+ T cells and hypothesized that the inflammatory process associated with SREAT may lead to increased permeability of the blood–brain barrier allowing for entry to, and persistence of, lymphocytes in the intrathecal space [20]. This is further supported by postmortem autopsies of patients with SREAT, in which diffuse lymphocytic infiltration around parenchymal vasculature has been identified, reflecting lymphocytic pleocytosis in the disease state [21].

The second case demonstrates a complex presentation in the context of the comorbidities the patient presented with. Overall, this case satisfies all diagnostic criteria laid out by Graus et al. with the exception of MRI findings and the reasonable exclusion of alternative causes. The patient exhibited myoclonus on physical examinations consistent with SREAT; however, she had preexisting cervical spinal stenosis that can also manifest as myoclonus. Further, brain MRI is expected to be normal or show nonspecific changes per Graus criteria; however, this patient had several traumatic brain injuries due to prior falls, making it difficult to distinguish findings due to prior injury versus current disease state. While these comorbidities may have contributed to her initial presentation, her lack of improvement with appropriate treatment for these conditions facilitated the consideration of other encephalopathies and the eventual diagnosis of SREAT after improved cognitive functioning after steroid treatment. This case highlights the importance of maintaining suspicion of SREAT even in the face of diagnostic complexity, especially following a traumatic presentation as it may obscure recognition. Further, these cases demonstrate how a finding of elevated antithyroid antibodies is not disease‐specific and lacks a consistent correlation with disease severity or treatment response. While many patients with SREAT present as euthyroid or only mildly hypothyroid, this condition can also present alongside profound hypothyroidism, as seen in the second case, emphasizing the need to maintain diagnostic consideration across different thyroid states (Table 1).

**TABLE 1:** Application of the Graus criteria for autoimmune encephalitis to our two clinical cases.

Graus criteria [13]	Case 1	Case 2
Encephalopathy with seizures, myoclonus, hallucinations, or stroke‐like episodes	Satisfies criteria	Satisfies criteria
Subclinical or mild overt thyroid disease (usually hypothyroidism)	Satisfies criteria	Satisfies criteria
Brain MRI normal or with nonspecific abnormalities	Leptomeningeal enhancement	Traumatic hematoma and hemorrhage
Presence of serum thyroid (thyroid peroxidase, thyroglobulin) antibodies	Satisfies criteria	Satisfies criteria
Absence of well‐characterized neuronal antibodies in serum and CSF	Satisfies criteria; however, pleocytosis in CSF is not common	Satisfies criteria
Reasonable exclusion of alternative causes	Satisfies criteria	Difficult to fully rule out contribution of comorbidities

## 5. Conclusions

Diagnosis of SREAT continues to rely on clinical judgment, systematic exclusion of other etiologies, detection of elevated antithyroid antibodies, and a therapeutic response to corticosteroids or other immunosuppressants. Knowing that timely treatment with immunosuppressive treatments results in favorable outcomes, a standardized diagnostic criterion, such as the Graus criteria, is highly beneficial for the early recognition of SREAT. However, this condition’s variable presentation and diagnostic workup, as evident in the described cases, should remind clinicians to carefully consider diagnostic criteria when exploring the possibility of SREAT. Meanwhile, clinicians should simultaneously appreciate that atypical findings may not rule out this condition, especially if the presentation is inconsistent with a known etiology. By sharing these atypical cases, we hope to provide insights into how to clinically use the Graus criteria to bring SREAT into the differential diagnosis while relying on clinical reasoning when making the official retrospective diagnosis.

Despite diagnostic challenges, early recognition and treatment can lead to favorable outcomes, even in severe cases. In this small series, both patients experienced substantial recovery, aligning with prior literature that timely initiation of immunosuppressive therapy often reverses neurological decline or significantly improves function. Overall, these cases contribute to the growing body of evidence that SREAT, although rare and elusive, should be considered in patients with subacute unexplained encephalopathy and elevated antithyroid antibodies. Careful evaluation, exclusion of alternative diagnoses, and prompt initiation of immunosuppressive therapy remain the cornerstones of management and are essential to achieving the best possible clinical outcomes [9, 13].

Future research on SREAT should aim to clarify the immunologic underpinnings of disease activity and treatment response. One direction could be to conduct a quantitative trend assessment of antithyroglobulin and thyroid peroxidase antibody titers before and after treatment to evaluate their utility as dynamic biomarkers of disease activity or therapeutic response. From a more foundational perspective, research around characterizing the cellular drivers of the immune response in SREAT, specifically around specific cytokines and B‐ and T‐cells, should be explored. This would be especially useful as current evidence points to this as a systemic autoimmune–driven process rather than a thyroid‐specific pathology [17]. Finally, identifying molecular and genetic predictors of corticosteroid responsiveness in patients with SREAT, such as HLA associations or neuroimmunogenetic profiles, could enable earlier and more finely tailored immunotherapies. Collectively, these directions would refine diagnostic and treatment modalities to enable more timely intervention.

## Funding

This study is funded internally by BMC Brighton, Department of Neurology.

## Consent

All the patients allowed personal data processing, and informed consent was obtained from all individual participants included in the study.

## Conflicts of Interest

The authors declare no conflicts of interest.

## Data Availability

The data that support the findings of this study are available from the corresponding author upon reasonable request.
